# Metaproteomics reveals parallel utilization of colonic mucin glycans and dietary fibers by the human gut microbiota

**DOI:** 10.1016/j.isci.2024.110093

**Published:** 2024-05-23

**Authors:** Grete Raba, Ana S. Luis, Hannah Schneider, Indrek Morell, Chunsheng Jin, Signe Adamberg, Gunnar C. Hansson, Kaarel Adamberg, Liisa Arike

**Affiliations:** 1 Department of Chemistry and Biotechnology, Tallinn University of Technology, 12618 Tallinn, Estonia; 2 Department of Medical Biochemistry and Cell Biology, University of Gothenburg, 41390 Gothenburg, Sweden; 3 SciLifeLab, University of Gothenburg, 41390 Gothenburg, Sweden; 4 Center of Food and Fermentation Technologies, 12618 Tallinn, Estonia

**Keywords:** Microbiome, omics

## Abstract

A diet lacking dietary fibers promotes the expansion of gut microbiota members that can degrade host glycans, such as those on mucins. The microbial foraging on mucin has been associated with disruptions of the gut-protective mucus layer and colonic inflammation. Yet, it remains unclear how the co-utilization of mucin and dietary fibers affects the microbiota composition and metabolic activity. Here, we used 14 dietary fibers and porcine colonic and gastric mucins to study the dynamics of mucin and dietary fiber utilization by the human fecal microbiota *in vitro*. Combining metaproteome and metabolites analyses revealed the central role of the *Bacteroides* genus in the utilization of complex fibers together with mucin while *Akkermansia muciniphila* was the main utilizer of sole porcine colonic mucin but not gastric mucin. This study gives a broad overview of the colonic environment in response to dietary and host glycan availability.

## Highlights

• Co-utilization of mucin and dietary fibers by microbiota was studied
• Metaproteomic analysis gave insight into consortia composition and metabolism
• *Akkermansia muciniphila* utilized the colonic mucin protein and its *O*-glycans
• Amino acids released from mucin affected the microbial metabolism

## Graphical abstract

**Figure:**
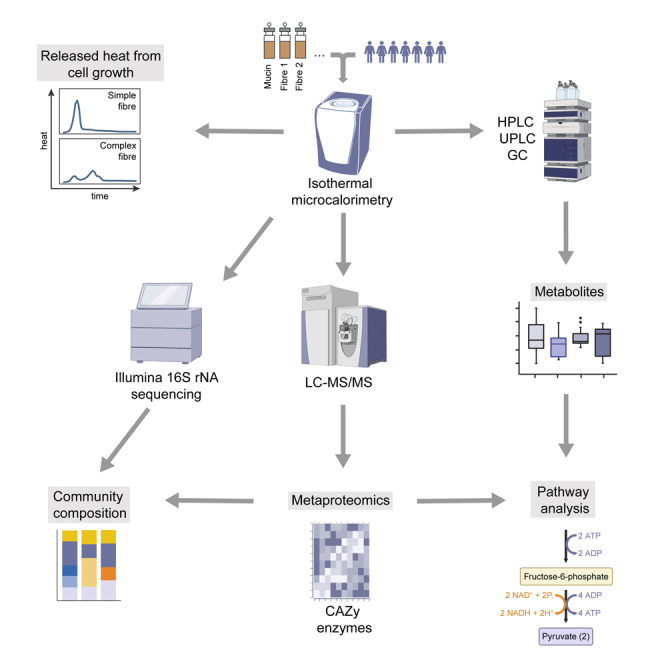

## Introduction

The human gut microbiota has an immense impact on human health and disease. These bacteria are well adapted to survive in the gastrointestinal tract due to their ability to utilize a wide range of dietary fibers and host glycans as a carbon source.^1^ The degradation of these saccharides by microbiota is catalyzed by different carbohydrate-active enzymes (CAZymes) such as glycoside hydrolases (GHs), polysaccharide lyases (PLs), carbohydrate esterases (CEs), and sulfatases.^2^^,^^3^^,^^4^ The downstream metabolism of the resulting monosaccharides by the bacteria provides the host with an amplitude of beneficial metabolites such as short-chain fatty acids (SCFAs) and biogenic amines.^5^^,^^6^^,^^7^

The microbiota in the gut colonizes the mucus layer that acts simultaneously as a protective barrier and an interaction site between the host and the bacteria.^8^ The major component of the colonic mucus layer is Mucin-2 (MUC2), a heavily *O*-glycosylated protein produced and secreted by epithelium goblet cells. The presence of gut microbes is required for normal epithelial development, mucins turnover, and the development of an impenetrable mucus barrier.^9^^,^^10^^,^^11^ However, some microbiota species can utilize mucins as a carbon source. Indeed, prolonged lack of dietary fibers and the resulting imbalances in the gut microbiota composition have been shown to promote the expansion of mucin-degrading bacteria and impaired mucus barrier function.^12^^,^^13^ Moreover, when the diet is low on fibers, some gut bacteria switch their metabolism to use mucin glycans by inducing gene expression of mucin-utilizing enzymes.^14^ It has been proposed that mucin degradation and the respective shifts in the microbiota metabolism have a crucial role in the development of diseases such as inflammatory bowel disease (IBD) and obesity.^12^^,^^15^ Yet, it remains poorly understood how the complex gut consortium utilizes colonic mucins and how this process is affected by the presence of dietary fibers.

Previous studies have disclosed how microbiota members have evolved to degrade plant cell wall polysaccharides.^16^^,^^17^ However, the understanding of the mechanisms behind colonic mucin utilization has been significantly limited due to the lack of an available substrate. Recent studies have been focused on characterizing specific enzymes from known species that can grow on porcine gastric mucin (PGM), such as *Akkermansia muciniphila*, *Bacteroides thetaiotaomicron*, *Bacteroides fragilis*, *Bacteroides caccae*, and *Ruminococcus gnavus*.^18^^,^^19^^,^^20^^,^^21^^,^^22^^,^^23^ However, the human gut microbiota is a complex consortium of hundreds of interacting species. It is likely that within the dynamic colonic environment some bacteria will rely on specific systems evolved for selfish utilization of glycans, whereas other members of the microbiota will share oligosaccharides and metabolites, allowing the growth of additional bacteria unable to access the complex glycans. Until now, these dynamic interactions have been addressed *in vitro* by using human fecal microbiota to determine the co-metabolism of dietary polysaccharides and PGM,^24^^,^^25^^,^^26^^,^^27^ but it remains unclear how colonic mucins impact the microbiota community metabolism. Recently it was shown that some bacterial species able to grow on PGM do not grow on porcine colonic mucin (PCM) *O*-glycans.^22^ Therefore, it is likely that differences in mucin *O*-glycosylation along the gastrointestinal tract^28^^,^^29^ have a major impact on the microbiota community. As the fermentation of dietary fibers happens mainly in the colon, the use of colonic mucins as co-substrate is required to mimic the physiological conditions. To understand the parallel utilization of mucins and dietary glycans, we cultivated human fecal microbiota on a panel of 14 dietary fibers with or without PGM or PCM. The co-fermentation of selected four fibers and PCM was further studied by metaproteomics as well as by metabolite analysis.

## Results

### The substrate determines the microbiota composition of the fecal consortium

**Figure 1:**
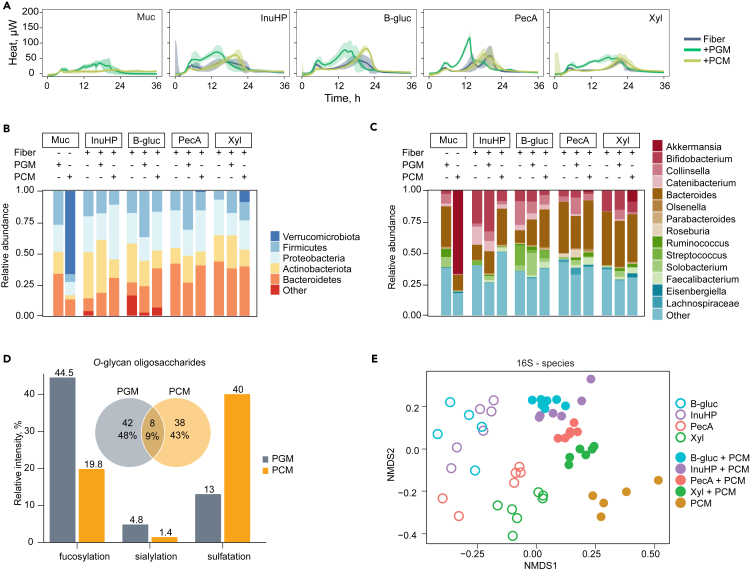
The substrate determines the microbiota composition of the fecal consortium (A) Heat evolution of substrate fermentation. The blue line represents the average microbial growth curve on the selected fiber, the green line on the selected fiber+PGM, and the yellow line on the selected fiber+PCM; ±95% confidence interval (CI) shown as a shaded area. *n* = 2–7. (B) Phylum-level community composition of the microbiota grown on the selected substrates based on 16S rRNA sequencing. Average relative abundances of the top five phyla. *n* = 2–7. (C) Genus-level community composition of the microbiota grown on the selected substrates based on 16S rRNA sequencing. Average relative abundances of the top 14 genera. *n* = 2–7. (D) The number and type of different *O*-glycan oligosaccharides detected in PGM and PCM. (E) Ordination plot of Bray-Curtis distances between microbial communities from cultivations on the selected substrates. Colors indicate the choice of dietary fiber, empty round dots represent samples from the cultivation of fiber, and filled round dots represent samples from the cultivation of fiber+PCM or sole PCM. Muc – mucin, InuHP – high-performance inulin, B-gluc – β-glucan, PecA – apple pectin, Xyl – xylan, PGM – porcine gastric mucin, PCM – porcine colonic mucin. See also Figure S1.

Fecal samples from seven healthy donors were pooled to generate a complex standardized inoculum that overcomes interindividual differences in microbiota. Isothermal microcalorimetry was used to study how these bacteria co-utilize different complex dietary and host glycans. Isothermal microcalorimetry measures the heat produced from metabolically active cells in hermetically sealed vials. This allows highly sensitive growth and biomass estimations in turbid environments without being affected by the precipitation of cells and high molar mass substrates.^30^^,^^31^ Community growth heat flows were constructed, where high and narrow peaks indicate fast degradation of the substrate, while low and wide peaks reflect slow and poor degradation (Figures 1A and S1A; Table S1). A panel of 14 dietary fibers were screened in this study. These were chosen to cover structurally variable polysaccharides that can be found in different dietary sources: arabinogalactan (AG), amylopectin (AP), β-glucan (B-gluc), κ-carrageenan (Car), furcellaran (Fur), galactooligosaccharides (GOSs), inulin (from dahlia [InuD], high-performance inulin [InuHP] and high-soluble inulin [InuHSI]), pectin (from apple [PecA] and citrus [PecC]), psyllium (Psy), xylooligosaccharides (XOSs), and xylan (Xyl) (Table S2). The growth medium was additionally supplemented with commercial PGM or in-house purified PCM to study the impact of host gastrointestinal glycans on dietary fiber fermentation.

The microbial composition that grew on each substrate was first evaluated with 16S rRNA sequencing. The more simple-structured fibers (AP, GOS, InuHSI, XOS) were rapidly fermented (Figure S1A) by fast-growing Proteobacteria and Actinobacteria (Figure S1B). These fibers contain linear chains of monosaccharides and, except for AP, have a degree of polymerization <10, making them easy to degrade by many bacteria. The fibers with a complex structure that contain more specific linkages and side chains, such as Fur, InuD, AG, PecC, Psy, InuHP, B-gluc, PecA and Xyl, were fermented slowly in multiple phases (Figures 1A and S1A). Although Car is a complex fiber, its fermentation profile and the resulting microbiota composition were similar to those of simple fibers (Figures S1A and S1B). This rapid growth of Car is likely due to the presence of short oligo- and monosaccharides as additives in the substrate. Like simple fibers, Fur degradation relied on Proteobacteria, while B-gluc, InuD, and InuHP favored the growth of Actinobacteria (Figures 1B and S1B). The fermentation of AG, PecC, Psy, PecA, and Xyl resulted in characteristically high proportions of Bacteroidetes (Figures 1B and S1B). This is consistent with studies showing that *Bacteroides* spp. are adapted to break down complex glycans.^32^ Similarly, mucins (PGM and PCM) are highly complex substrates containing a variety of linkages which cannot be fully degraded by a single species, as is demonstrated by slow degradation of mucin by a diverse community (Figures 1A–1C). The co-fermentation of fibers with mucin resulted in prolonged growths and increased abundances of Bacteroidetes and Firmicutes compared to the fermentation of sole fibers (Figures 1A, 1B, S1A, and S1B). The co-fermentation of Xyl, Car, Fur, and Psy with PGM resulted in similar growth curves as sole PGM (Figures 1A and S1A). However, whereas Xyl+PGM promoted the growth of *Bifidobacterium* (Figures 1B and 1C), the fermentation of Car+PGM, Fur+PGM, and Psy+PGM resulted in consortia similar to that of sole PGM (Figures S1B and S1C), suggesting that mucin was preferentially fermented over the insoluble fiber component. PGM promoted the growth of *Ruminococcus* and *Bacteroides*, two genera that contain known mucin degraders,^33^^,^^34^ and *Faecalibacterium* who has been shown to be in a syntrophic relationship with mucin degraders^35^ (Figures 1C and S1C). The additional glycans from PGM also lowered the abundances of some simple fast-growing genera, especially *Escherichia/Shigella* (included in the “other” genera in Figure 1C). The growth of this genus is known to be artificially boosted in batch culture conditions where there is no control over substrate availability, resulting in overgrowth of such taxa from mixed culture inoculums.^36^^,^^37^ Despite none of the single-fiber fermentations reaching the microbial diversity of the inoculum (Figures S1D and S1E), complex fibers helped to maintain more diverse consortia compared to the fast-fermented glycans (AP, GOS, and InuHSI) (Figure S1E). Furthermore, supplementing the cultures with either of the mucins increased the overall microbial diversity when compared to the respective fiber only (Figure S1E).

As the two sources of mucin lead to distinct microbial communities, we carried out a comparison of in-house-extracted PCM and commercially available PGM to understand the differences between these substrates. Consistent with the literature,^38^ PCM contained mainly MUC2, while PGM was a mixture of MUC5AC and MUC6 (Figure S1F). Mass spectrometry analysis of released *O*-glycans from PGM and PCM samples identified only 9% of shared *O*-glycans as well as distinctly different modifications, with PGM being mainly fucosylated, while PCM was highly sulfated (Figure 1D). Moreover, our analysis suggested PGM to be severely hydrolyzed (Figures S1G and S1H) which is likely to lead to a faster fermentation of this substrate. Commercial PGM has also been shown to contain various contaminants, which can support the growth of additional species, besides mucin utilizers.^39^^,^^40^ Together, these results indicate that commercial PGM is structurally different from in-house purified PCM which is a more physiologically relevant mucin source to address the impact of host glycans on colonic microbiota communities.

To further understand how the metabolism of complex fibers and colonic mucin affects the microbiota, B-gluc, PecA, and Xyl were selected for an in-depth study based on their distinctly different molecular structures that require specific degradative enzymes encoded in different bacteria. A known prebiotic, InuHP, was included as an easily fermentable control. The community grown on sole PCM was dominated by *Akkermansia*, a known slow-growing mucin-utilizing bacterium (Figure 1C). Surprisingly, PGM and its combinations with fibers did not promote growth of *Akkermansia* (Figure 1C). Compared to fiber+PGM, the fiber+PCM combinations increased the abundances of gut commensals *Bacteroides* and *Faecalibacterium* and decreased the abundances of an opportunistic pathogen *Solobacterium* and a pathobiont *Collinsella* (Figure 1C). Altogether, the microbial growth curves and consortia composition analyses suggest that the co-metabolism of dietary fibers and mucins promotes the growth of different microbiota communities. While the degradation of each of the four fibers resulted in distinct communities, the addition of PCM reduced the dissimilarity (Figure 1E).

### Metaproteomic analysis of community composition

**Figure 2:**
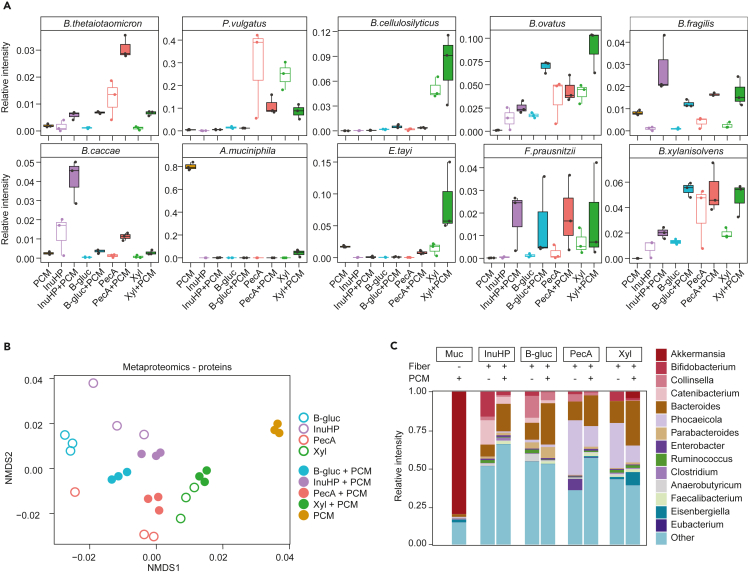
Metaproteomic analysis of community composition (A) Boxplots showing changes in microbial abundances (relative intensity of unique peptides) grown on the selected substrates. Colors indicate the choice of substrate, empty boxes represent samples from the cultivation of fiber, and filled boxes represent samples from the cultivation of fiber+PCM or sole PCM (*n* = 3). Kruskal-Wallis multiple comparisons with Benjamini Hochenberg corrections can be found in Table S1. Muc – mucin, InuHP – high-performance inulin, B-gluc – β-glucan, PecA – apple pectin, Xyl – xylan, PGM – porcine gastric mucin, PCM – porcine colonic mucin. See also Figures S2 and S3. (B) Ordination plot of Bray-Curtis distances between microbial proteins from cultivations on the selected substrates. Colors indicate the choice of dietary fiber, empty round dots represent samples from the cultivation of fiber, and filled round dots represent samples from the cultivation of fiber+PCM or sole PCM. (C) Genus-level community composition of the microbiota grown on the selected substrates based on unique peptides from metaproteomic analysis. Average relative intensity of the top 14 genera. *n* = 3. See also Figures S2 and S3.

Metaproteomic search against an in-house-constructed database consisting of 290 bacterial taxa as well as human and pig sequences was used for a deeper species-level separation of the community composition. The database was constructed by combining *de novo* search with taxa-specific peptide assignments. We and others propose this approach as an alternative for combining databases from metagenomic assemblies.^41^^,^^42^ We identified 110,115 distinct peptides corresponding to 21,750 protein groups, with a median of 19,872 peptide sequences (7,896–33,500) and 6,005 (2,704–8,767) protein groups for each sample (Figures S2A and S2B; Table S3). After combining triplicate experiments, we identified a median of 33,512 (16,759–46,516) peptides corresponding to 8,394 (4,827–11,773) protein groups per sample group (Figures S2A and S2B). Human and pig proteins constituted on average 2% and 1%, respectively, of all the identifications. Metaproteomics allowed us to separate *Bacteroides* species that were unclassified by 16S rRNA sequencing, *Bacteroides acidifaciens*, *Bacteroides finegoldii*, *Bacteroides ovatus*, and *Bacteroides xylanisolvens* (Figure S3A), as well as to identify species such as *Bacteroides cellulosilyticus* (Figure 2A) which were unidentified by 16S rRNA sequencing. To overcome the overlapping peptide sequences between species, we carried out the analysis on unique peptides for each species. The addition of PCM changed the composition of fecal microbiota (Figure 1E) as well as proteins that were expressed (Figure 2B), while reducing dissimilarity between conditions, likely caused by the need for highly specialized species and enzymes to break down mucin glycans. Metaproteomic analysis revealed distinct substrate-specific clustering of samples based on the identified proteins (Figure 2B) as well as the species preference toward each glycan source (Figures 2A and S3B).

InuHP fermentation was characterized by high abundances of *Bifidobacterium* and *Catenibacterium* species (Figures 2C and S3B, for multiple comparisons see Table S1). B-gluc increased the abundances of *Collinsella*, *Parabacteroides*, *Streptococcus*, and *Solobacterium*, in addition to butyrogenic *Anaerobutyricum hallii* (Figures 2C and S3B). The complex fibers pectin and xylan led to an overall expansion of *Bacteroides* and *Phocaeicola* species (Figure 2C). Pectin promoted the growth of *B*. *thetaiotaomicron* and *Phocaeicola vulgatus* (Figure 2A), while xylan notably promoted the growth of *B. cellulosilyticus*, *B*. *ovatus*, and *P. vulgatus* (Figure 2A), species known to be able to degrade these complex polysaccharides.^17^^,^^43^^,^^44^^,^^45^^,^^46^^,^^47^ Xylan was the only fiber which, in combination with PCM, promoted the growths of *A*. *muciniphila* and *Eisenbergiella tayi* (Figure 2A). The abundances of some *Bacteroides* and *Phocaeicola* species were affected by the presence of mucin. The abundance of *P. vulgatus* (Figure 2A) and *Phocaeicola dorei* (Figure S3B) was higher without mucin, while *B*. *thetaiotaomicron*, *B*. *fragilis*, and *B*. *caccae* abundance increased when fibers were co-fermented with PCM (Figure 2A). *B. ovatus*, *Faecalibacterium prausnitzii*, *B*. *xylanisolvens*, and *Parabacteroides merdae* had higher abundance when fiber was co-fermented with PCM, but they were not able to grow on sole PCM (Figures 2A and S3B).

### Degradation mechanisms of mucin and fiber by human gut microbiota

In addition to compositional analysis, metaproteomics delivers also functional information of the active microorganisms. Therefore, we next identified the enzymes involved in degrading complex glycans. More than 21,000 protein groups were identified, out of which around 5% were mapped to Carbohydrate-Active enZYmes Database (CAZy, cazy.org)^3^ containing proteins involved in polysaccharide recognition and degradation (Figure S3C). Additionally, SulfAtlas database of sulfatases^2^^,^^4^ was used to identify sulfatases.

**Figure 3:**
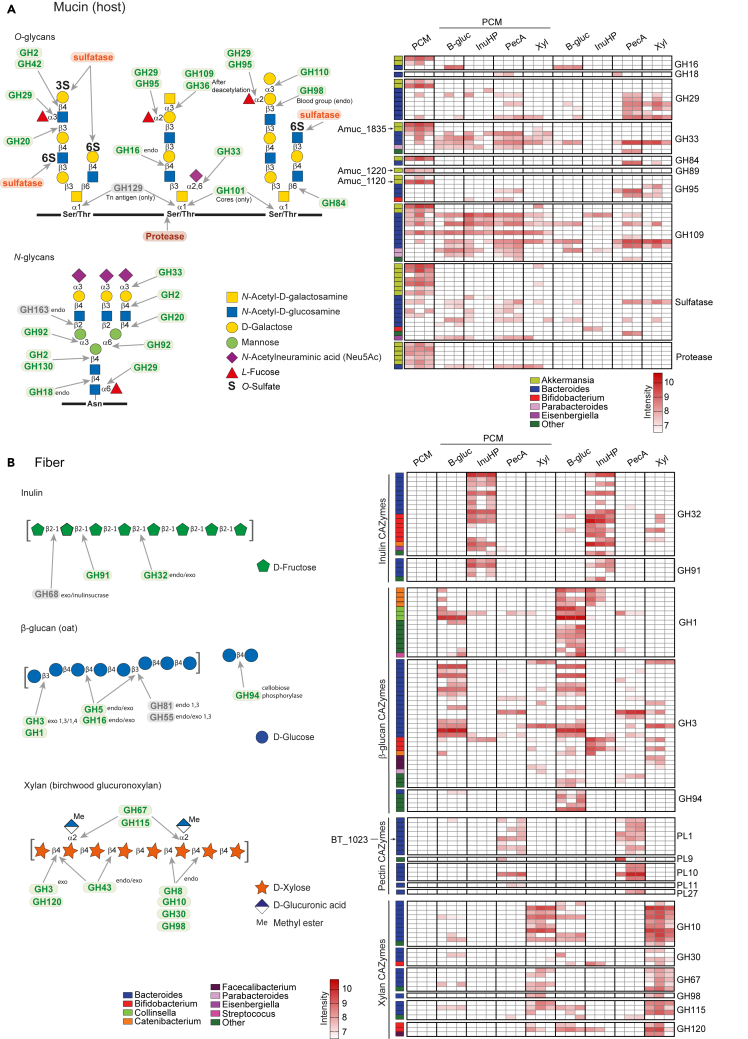
Degradation mechanisms of mucin and fiber by human gut microbiota (A) Structures of mucin *O*- and *N*-glycans and heatmap of detected CAZymes targeting the respective linkages. (B) Structures of inulin, β-glucan, and xylan and heatmap of CAZymes targeting the respective linkages. Enzymes grouped by CAZyme families are shown as MS intensity of the protein. For each protein, the respective bacterial genus is displayed by a color code on the left side. InuHP – high-performance inulin, B-gluc – β-glucan, PecA – apple pectin, Xyl – xylan, PGM – porcine gastric mucin, PCM – porcine colonic mucin, GH – glycoside hydrolase, PL - polysaccharide lyase. See also Figures S2 and S4.

The CAZymes detected from the degradation of sole PCM were mainly from *A*. *muciniphila* (Figures 3A and S4A), a result consistent with the high abundance of this bacterium on sole PCM, as shown by both 16S rRNA sequencing and metaproteomic analysis (Figures 1C, 2A, and 2C). In addition to enzymes annotated to CAZymes, we also detected 36 *A. muciniphila* proteins with unknown function that could potentially include additional enzymes with mucin-utilization activity (Table S3). When PCM was co-fermented with fiber, we identified CAZymes from *Bacteroides* species known to be able to utilize mucin, such as *B. caccae*, *B*. *fragilis*, and *B. thetaiotaomicron* (Figures 3A and S4A). These CAZymes belong to families known to contain mucin glycan-degrading enzymes, such as GH2 (β-galactosidases), GH20 and GH84 (β-*N*-acetylglucosaminidases), GH29 and GH95 (α-fucosidases), GH89 (α-*N*-acetylglucosaminidases), and GH33 (sialidases) (Figures 3A and S4A). Specifically, the enzymes Amuc_1835 (GH33), Amuc_1120 (GH95), Amuc_0290 (GH2), and Amuc_1220 (GH89), detected on PCM (Figure 3A), have been shown to be critical for the growth of *A. muciniphila* on PGM.^48^ We detected multiple sulfatases from *A. muciniphila*, *B. fragilis*, and *B. ovatus*, suggesting that these bacteria could have a critical role in removing the capping sulfate groups from mucin *O*-glycans (Figure 3A). Surprisingly, the α/β-*N*-acetylgalactosaminidases from mucin-specific family GH109 were also detected in some of the fiber samples in the absence of PCM, suggesting that this family of enzymes might contain proteins targeting additional linkages found in dietary fibers. We detected several proteases that can potentially cleave the mucin backbone: Amuc_1119 OgpA,^49^ Amuc_1434,^50^ Amuc_1438, Amuc_2001 M60,^51^ Amuc_1514 M98, and BT_4244 M60^52^ (Figure 3A; Table S3).

The degradation of InuHP was associated with GHs from families 32 and 91, known to be populated only with fructose-active enzymes^3^ (Figure 3B). These GHs were expressed mostly by *Bacteroides* and *Bifidobacterium* species, consistent with the high abundance of these bacteria by 16S rRNA sequencing (Figure 1C) and metaproteomics (Figure 2C).

B-gluc degradation relied mainly on enzymes from families GH1, GH3, and GH94 that cleave β-glucose linkages (Figure 3B). The 16S rRNA sequencing (Figure 1C) and metaproteomics (Figures 2C and S3B) showed an increase in *Collinsella* and *Streptococcus* abundances. GH1 enzymes from *Collinsella* were among the most expressed CAZymes on B-gluc while only one CAZyme (GH1) was detected from *Streptococcus* (Table S3), suggesting that *Collinsella* has a key role in B-gluc degradation within the community while *Streptococcus* is likely to rely on other species for cross-feeding. Additionally, we identified multiple CAZymes from *Catenibacterium* and *Bacteroides* species that are specifically associated with B-gluc utilization, suggesting that these bacteria can have a role on this polysaccharide degradation (Figure 3A).

We detected the most proteins involved in carbohydrate metabolism from samples of PecA degradation, reflecting its high structural complexity (Figure S4C). The CAZymes detected from PecA fermentation were mostly from various *Bacteroides*/*Phocaeicola* species *(B. ovatus*, *B. thetaiotaomicron*, *P. vulgatus*, and *Phocaeicola sartorii*) (Figures 3B and S4B). Pectin is composed of three main fractions: rhamnogalacturonan (RG) I and II and homogalacturonan (Figure S4C). The metaproteome analysis of microbiota grown on PecA revealed multiple CAZymes associated specifically with the degradation of these polysaccharides, such as PLs of families 1, 9, 10, and 11 (Figure 3B) and polygalacturonases (GH28), arabinofuranosidases (GH43 and GH51 [shared with xylan]), unsaturated rhamnogalacturonyl hydrolases (GH105), and rhamnosidases (GH106) (Figure S4B). An enzyme from PL27, a family populated with l-rhamnose-α-1,4-d-glucuronate lyase active on AG, was also associated with PecA degradation^53^ (Figure 3B). RG II, the most complex polysaccharide in nature, required additional enzymes for full degradation, such as aceric acid hydrolases (GH127), rhamnosidases (GH78), Kdo hydrolase (GH33, a family containing mucin active enzymes), and α-galactosidases (GH95, a family known to have mucin and xylan active enzymes) (Figures 3A and S4B). Of notice, we detected a PL1 enzyme (BT_1023) that was previously described as critical in RG II degradation by *B. thetaiotaomicron*^54^ (Figure 3B). From cultures grown on PecA, we also found some uncharacterized proteins that demonstrated strong substrate specificity toward PecA, such as BT_3240 (SusC homolog), BT_3241 (SusD homolog), and BT_3242 (unknown protein) (Figure S4B).

The degradation of Xyl was mainly carried out by β-xylanases, α-glucuronidases, arabinofuranosidases, and β-xylosidases belonging to families GH8, GH10, GH30, GH43, GH51, GH67, GH98, GH115, and GH120 (Figures 3B and S4B). *Bacteroides* enzymes account for most of the GHs associated with Xyl degradation. Interestingly, several of these enzymes are found in polysaccharide utilization loci (PULs) similar to the previously characterized *B. ovatus* xylan PULs.^43^ Overall, by mapping the proteins from metaproteomic analysis to enzymes from the CAZy and SulfAtlas databases, we were able to identify representatives for most of the CAZyme families described in the literature for each studied glycan.

### Analysis of downstream metabolism of mucin and fiber by the fecal consortia

The central metabolism of consortia grown on fibers with or without PCM was elucidated by measuring extracellular metabolites such as SCFAs, biogenic amines, amino acids, and gases from the end of the growth by liquid and gas chromatography. Additionally, the bacterial metabolism was evaluated via metaproteome analysis by mapping the identified peptide sequences to public NCBI database (blast.ncbi.nlm.nih.gov) and counting the enzyme copies identified for each central metabolic pathway reaction (Table S4).

**Figure 4:**
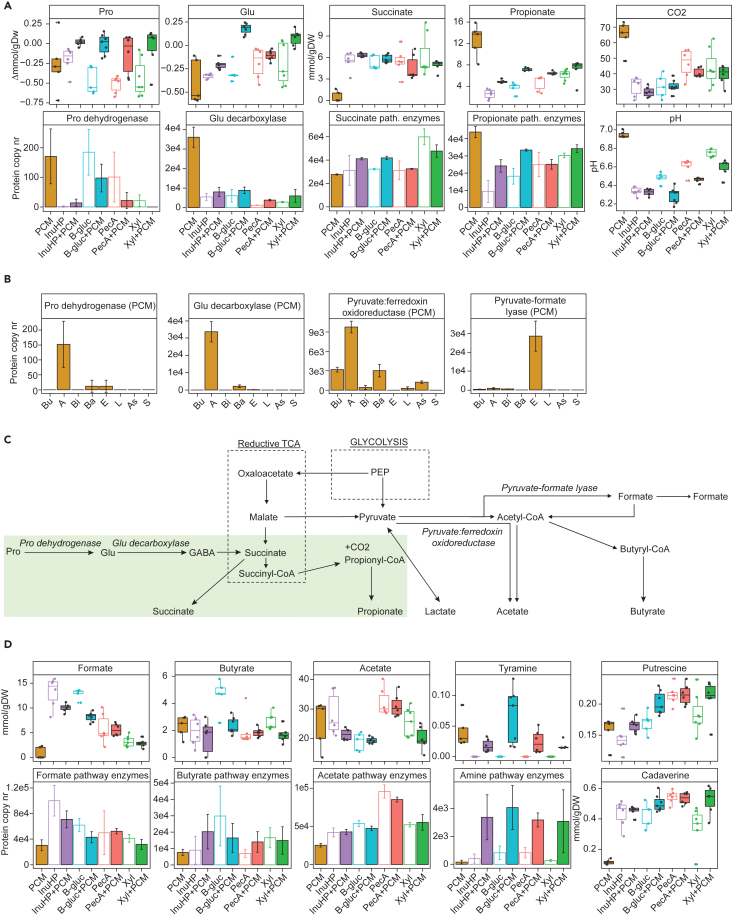
Analysis of downstream metabolism of mucin and fiber by the fecal consortia (A) Boxplots representing the consumption of amino acids and the concentrations of measured metabolites (mmol/gDW) and pH (*n* = 5–7) and barplots representing average (±SD) enzyme counts per condition (*n* = 3) for reactions related to specific metabolite synthesis. Colors indicate the choice of substrate, empty boxes and bars represent samples from cultivation of fiber, and filled boxes and bars represent samples from the cultivation of fiber+PCM or sole PCM. Kruskal-Wallis multiple comparisons with Benjamini Hochenberg corrections can be found in Table S1. (B) Average (±SD) enzyme counts per bacterial group for reactions related to specific metabolite synthesis. *n* = 3. To simplify the metaproteomic assessment of bacterial crosstalk, the bacteria in consortia were divided into eight metabolic groups based on information acquired from phylogenetic data in NCBI database and genomic annotations: 1) butyric (Bu) – butyrate and 1,2-propanediol producers, 2) akkermansia (A) – mucolytic *A. muciniphila*, 3) bifido (Bi) – lactate and acetate producers, 4) bacteroides (Ba) – propionate/succinate and acetate producers, 5) enterobacteria (E) – lactate, succinate and acetate producers, 6) lachnoclostridia (L) – mucolytic, formate and 1,2-propanediol consumers, 7) asaccharolytic (As) – lactate and amino acid degraders, and 8) succinivorans (S) – succinate consumers (see STAR Methods). (C) Schematics of metabolic pathways to produce main bacterial metabolites. (D) Boxplots representing the concentrations of measured metabolites (mmol/gDW, *n* = 5–7) and barplots representing average (±SD) enzyme counts per condition (*n* = 3) for reactions related to specific metabolite synthesis. Colors indicate the choice of substrate, empty boxes and bars represent samples from the cultivation of fiber, and filled boxes and bars represent samples from the cultivation of fiber+PCM or sole PCM. InuHP – high-performance inulin, B-gluc – β-glucan, PecA – apple pectin, Xyl – xylan, PGM – porcine gastric mucin, PCM – porcine colonic mucin.

The detection of proteases capable of cutting mucin backbone from *A. muciniphila* and *B. thetaiotaomicron* grown on sole PCM (Figure 3A) suggested that these bacteria were partially utilizing the MUC2 protein, in addition to its *O*- and *N*-glycans. Indeed, consumption of proline (Pro), accompanied by elevated levels of proline dehydrogenase (Figure 4A), was detected mainly from *A. muciniphila* grown on sole PCM (Figure 4B). The MUC2 mucin domain consists of repeating units of proline, threonine, and serine. Released proline can be used to produce glutamate (Glu), through its conversion by proline dehydrogenase (Figure 4C). Glutamate itself is a precursor to produce γ-aminobutyric acid (GABA) (Figure 4C), an intermediate metabolite of colonic microbiota. Indeed, there was a high concentration of glutamate decarboxylase from *A. muciniphila* grown on sole PCM (Figures 4A and 4B). However, no GABA itself was detected from the culture supernatant. Similarly, succinate concentrations measured from the end of the fermentations were near zero, while the metaproteome analysis showed a highly active succinate synthesis pathway (Figure 4A). These data suggest an efficient conversion of GABA into succinate and the following conversion into propionate and CO_2_—the main metabolites of *A. muciniphila* which were both detected at elevated concentrations in the supernatant of sole PCM fermentation (Figure 4A). The metaproteomic analysis showed *A. muciniphila* to use pyruvate:ferredoxin oxidoreductase for the production of acetyl-coenzyme A (CoA) (Figure 4B). This is an NADH-releasing alternative to pyruvate-formate lyase pathway (Figure 4C). NADH itself is needed for the synthesis of propionate. Moreover, whereas the fermentation of glycans leads to lowering of the pH due to the release of SCFAs, the pH of spent medium from sole PCM fermentation was almost neutral (Figure 4A), implying the release of peptides and amino acids that were fermented into ammonia, increasing the pH.^55^ Using rough estimations, the added 0.5 g/L of mucin, with *ca.* 10 mol-% of proline, a maximum of 0.5 mM of propionate could have been produced from PCM, accounting for 5%–10% of the total propionate produced from sole PCM fermentation.

Inulin is a fructose polymer from which monomers are directed into central metabolism via glycolysis. The downstream metabolism of those fructose monomers results in an elevated production of formate, as was seen by both its high concentration and the number of enzymes related to formate synthesis on samples of InuHP degradation (Figure 4D). Similarly, the glucose monomers of β-glucan are directly channeled into glycolysis. Β-gluc fermentation was characterized by high concentration of butyrate and high number of butyrate synthesis enzymes, although both decreased during parallel fermentation with PCM (Figure 4D). Pectin degradation was accompanied by elevated production of acetate (Figure 4D), which can be directly released from the substrate as a result of bacterial esterase activity (RG I and II, Figure S4C), in addition to being produced by bacterial central metabolism (Figure 4C). As a response to the increased release of SCFAs from fiber and mucin co-fermentation, the consortia co-utilizing these substrates produced more cadaverine, putrescine, and tyramine (Figure 4D), to balance the environmental pH (Figure 4A). Together, these data show that the dietary glycans are degraded by a complex community of bacteria and the presence of mucin affects these metabolic pathways.

## Discussion

A panel of fourteen dietary fibers was incubated with a complex fecal inoculum with and without mucin to study how the host glycans affect the fermentation of different dietary fibers. Dietary fibers are crucial for maintaining a diverse gut microbiota that encodes a unique enzymatic capacity. Specialized bacteria are needed to degrade complex polysaccharides undigestible by the host. Meanwhile, regular consumption of dietary fibers is necessary to balance the numbers of fiber degrading and mucin-utilizing bacteria to keep the intestinal homeostasis and to ensure that the epithelium remains protected by the mucus layer.^12^^,^^15^ This delicate balance is maintained by the complexity and variety of linkages found in fiber and host glycans which ensures that the degradation takes place as an orchestrated co-operation of multiple different species.^25^^,^^47^^,^^56^^,^^57^^,^^58^^,^^59^ Here, we demonstrated that the polysaccharide structure and complexity determined the microbiota composition with the more complex glycans supporting more diverse consortia. Additionally, we have shown that the source of mucins has a major impact on microbiota growth and composition. It has been shown that glycosylation is variable between species and along the gastrointestinal tract. In this study, we describe key differences in glycosylation of PGM and PCM that impact microbiota growth. Therefore, we suggest that PCM is a more physiologically relevant source of host glycans and this substrate should be preferentially used in future colonic microbiota studies.

The use of dietary fibers as prebiotics requires that they elicit similar effects in broad populations; however, this impact is hampered by the interindividual variety of microbiota composition. It has been shown that the degradation of some dietary fibers can have very specific geographical and cultural limitations. For example, some enzymes that are required for the degradation of algal polysaccharides are found only in populations whose diet regularly includes algae.^60^^,^^61^ In this study, we observed a similar effect with algae fibers Car and Fur, as well as plant-based Psy, which are not part of the North-European diet and were not digested by the pooled local microbiota. Nevertheless, recent studies have shown that, despite the large differences in gut microbiota communities, more complex-structured dietary fibers can induce similar changes in consortia of different people.^57^^,^^58^^,^^62^ We demonstrated that different fibers changed the composition of pooled seven individual’s fecal microbiota as well as proteins that were expressed. Furthermore, the addition of PCM reduced dissimilarity. This is likely driven by highly specialized species and enzymes that are required to break down mucin glycans. Therefore, the presence of mucin glycans has an important role in defining the microbiota community, and the role of these host glycans should be considered in further studies aiming to identify prebiotics. Previous studies comparing single fibers and fiber mixtures show the latter to maintain microbiota diversity more effectively.^63^ In this study, we have shown the impact of the presence of mucin in microbial metabolism of single dietary glycans. Future studies are necessary to address the impact of host glycans in combination with complex fiber mixtures.

Colonic microbiota is a complex consortium where each species has its role. With metaproteomic analysis, it was possible to obtain a species-level understanding of the consortia composition. We demonstrated the central role of different *Bacteroides* species in the complex consortia for glycan degradation. Previous studies with monocultures have shown this genus to be particularly well adapted to utilizing complex polysaccharides due to the high number of CAZymes encoded by these bacteria.^16^^,^^22^^,^^32^^,^^46^^,^^64^ Additionally, metaproteomics allowed us to explore bacterial metabolism. We were able to map multiple CAZymes from different species grown on specific fibers and mucin. In previous studies these enzymes had been studied mostly in pure cultures. By deploying a metaproteomic approach we have shown that these enzymes can be identified in complex cultures. In addition to identifying previously described CAZymes with known functionality, we also found some uncharacterized proteins with strong substrate specificity suggesting that these proteins are likely to have a role in the utilization of these specific glycans. Of relevance, BT3240, BT3241, and BT3242 were specifically detected in cultures grown on pectin. These proteins are encoded in *B*. *thetaiotaomicron* PUL 52 (*bt3235-3244*), which encodes 6 proteins of unknown function and a SusC/D-like pair. Therefore, it is possible that this PUL is associated with *B*. *thetaiotaomicron* utilization of pectins.

Most strikingly, sole PCM promoted the growth of mucolytic *A*. *muciniphila*, a potential next-generation probiotic species due to its unique properties.^33^^,^^65^^,^^66^^,^^67^^,^^68^ This slow-growing bacterium has difficulties competing with other bacteria in conditions where there is no control over the limiting substrate,^27^ such as the case with batch cultivation. In the medium supplemented with commercial PGM, which we show that is partially hydrolyzed and known to contain easily fermentable contaminants, such as glycosaminoglycans, amino acids, peptides, lipids, and even metals,^39^^,^^40^ *A. muciniphila* was not able to grow in the complex consortium. However, when in-house purified PCM was used as a mucin source, *A. muciniphila* dominated the culture and expressed a multitude of CAZymes related to mucin degradation. This was even more surprising, considering previous studies that have shown *A. muciniphila*’s inability to grow on the *O*-glycans released from PCM in a monoculture.^22^ Moreover, the analyses of the metaproteome, CAZymes, and metabolites indicate that *A. muciniphila* grown on sole PCM was even partially utilizing the MUC2 protein itself. These findings could be either complex consortium specific or due to the use of intact full PCM and warrant a further investigation. Similarly, we found 36 proteins with unknown function from *A. muciniphila* that were upregulated on growth on sole PCM and are suitable candidates for future studies aiming to discover new activities encoded by gut commensals in order to understand how the balance between microbiota and glycans is maintained.

In addition to energy production, the probiotic effect of microbiota manifests itself through the release of host-beneficial metabolites such as SCFAs which are produced via glycan fermentation. Mucins, however, introduce another source of nutrients to the microbiota: peptides and amino acids. A multitude of gut bacteria can catabolize basic amino acids into amines with various biological effects on the host. Arginine is catabolized into putrescine which enhances epithelial cell proliferation.^69^^,^^70^ Lysine is used to produce cadaverine, which has been shown to support cell proliferation and gut barrier function.^6^^,^^71^ However, the host-beneficial effects need further confirmation as elevated concentrations of cadaverine have also been associated with ulcerative colitis.^72^ Similarly, the aromatic amino acid tyrosine can be converted into tyramine which has been shown to exert toxicity toward epithelial cells.^73^ In addition to the direct effect of individual metabolites to the host, these also contribute to maintaining the intestinal homeostasis through pH regulation. The release of SCFAs decreases the pH, while free peptides and amines increase it. At the same time, pH is a mediator of microbiota structure and function.^27^^,^^74^^,^^75^ This fine-tuning between the diet, the bacteria, and the host forms a feedback loop where the availability of nutrients and signals from the host determine microbiota composition. Next-generation probiotic species are considered to have the potential to regulate these systems through their specialized metabolism. Of notice, in this study, xylan was the only fiber which, in combination with PCM, promoted the growths of *A. muciniphila* and *E.* *tayi.* Little is known of the butyrogenic *E. tayi*.^76^ However, its concomitant growth with *A. muciniphila* together on xylan+PCM is interesting and warrants further investigation.

Conclusively, this study demonstrates new insights into co-utilization of colonic mucin and dietary fibers by the gut microbiota. The results of microbial growth and metabolism on various dietary and host glycans together with the metaproteomics form a valuable database to support future studies of colonic microbiota.

### Limitations of the study

This study showed the impact of presence of mucin on the metabolism of single dietary fibers. Previous studies comparing single fibers and fiber mixtures have shown the latter to be more effective in maintaining diverse microbial communities. Moreover, a real human diet consists of multiple different fibers, expanding the potential inter-species crosstalk. Therefore, future studies are needed to understand how the metabolism of fiber mixtures is affected by the presence of mucin. Additionally, the communities growing on each culture and their metabolism could be studied in more detail by using advanced cultivation methods. In this study batch cultivation was used to keep the working volumes low and allow screening of multiple substrates and in-house purified PCM. Methods such as continuous cultivation in bioreactors, which allow control over substrate availability, could offer additional insight into consortia development and metabolism.

## STAR★Methods

### Key resources table

**Table:** 

REAGENT or RESOURCE	SOURCE	IDENTIFIER
**Biological samples**		

Mucin from porcine colon	this paper	PCM
Slurry of human volunteer donor fecal samples	this paper	Inoc
MUC2 purified from LS174T	Recktenwald and Hansson, 2016^77^	hMUC2

**Chemicals, peptides, and recombinant proteins**		

Arabinogalactan from larch wood	Sigma-Aldrich	Cat#10830
Amylopectin from maize	Sigma-Aldrich	Cat#10120
Beta-glucan from oats	UNDERSUN BIOMEDTECH	N/A
Kappa-carrageenan, sulphated plant polysaccharide from *Eucheuma cottonii*	Sigma-Aldrich	Cat#C1263
Furcellaran, extracted from algae *Furcellaria lumbricalis* (*Gigartinales*)	Est-Agar AS	N/A
Galactooligosaccharides, DP 2-10	Friesland Campina	N/A
Inulin from dahlia tubers	Sigma-Aldrich	Cat#I3754
High performance inulin, DP > 23, 0.1% monosaccharides	Beneo Orafti	N/A
High soluble Inulin HSI, DP 2–8, 11% monosaccharides	Beneo Orafti	N/A
Pectin from apple	Sigma-Aldrich	Cat#93854
Pectin from citrus peel, Galacturonic acid >74%	Sigma-Aldrich	Cat#P9135
Psyllium (Carepsyllium), from *Plantago ovatas*, 80%	Caremoli	N/A
Xylooligosaccharides, 80%	Anhui Elite Ind Co	N/A
Xylan from beechwood	Sigma-Aldrich	Cat#X4252
Mucin from porcine stomach, Type III	Sigma-Aldrich	Cat#M1778
HiMark™ Pre-stained Protein Standard	Thermo Fisher Scientific	Cat#LC5699

**Critical commercial assays**		

PureLink™ Microbiome DNA Purification Kit	Thermo Fisher Scientific	Cat#A29790
Nextera XT DNA Library Preparation Kit	Illumina	Cat#FC-131-1024
Qubit™ dsDNA HS Assay Kit	Thermo Fisher Scientific	Cat#Q32851
Qubit™ dsDNA BR Assay Kit	Thermo Fisher Scientific	Cat#Q32850
Acquity; AccQ·Tag™ Ultra Derivatization Kit	Thermo Fisher Scientific	Cat#NC1795497

**Deposited data**		

16S rRNA sequencing data	NCBI Sequence Read Archive	PRJNA873127
Metaproteome dataset	ProteomeXchange Consortium	PXD035981
Proteome dataset	ProteomeXchange Consortium	PXD036037
Gycomic MS raw files	GlycoPOST	GPST000280

**Software and algorithms**		

BION-meta	McDonald et al., 2016^78^	https://github.com/nielsl/mcdonald-et-al
PEAKS Studio (version 8.5)	Bioinformatics Solutions Inc^79^	version 8.5
ProteoClade	Mooradian et al., 2020^41^	N/A
MaxQuant	Cox et al., 2008^80^	versions 1.4 and 1.6.11.0
dbCAN2	Zhang et al., 2018^81^	http://bcb.unl.edu/dbCAN2/blast.php
RStudio	RStudio, Inc.	version 1.2.5001

**Other**		

Dialysis tubing, 12,4 kDa cut-off, 99.99% retention	Sigma-Aldrich	Cat#D0530
Nanosep™ centrifugal devices with Omega™ membrane 30K, red	Pall Life Sciences	Cat#OD030C35
C18-StageTip filters	Rappsilber et al., 2007^82^	N/A
Reverse-phase column (150 × 0.075 mm inner diameter, C18-AQ 3 μm)	in-house packed	N/A
Custom metaproteomics database	this study	N/A
C18 ZipTip	Schulz et al., 2002^83^	N/A
Polar column (100 × 0.25 mm inner diameter, porous graphite 5 μm)	in-house packed	N/A
Invitrogen™ Novex™ Tris-Glycine Mini Protein Gels, 4–20%, 1.0 mm, WedgeWell™ format	Thermo Fisher Scientific	Cat#15492485
Amicon® Ultra-0.5 3 kDa cut-off filter	Merck	Cat#UFC5003

### Resource availability

#### Lead contact

Further information and requests for resources and reagents should be directed to and will be fulfilled by the lead contact, Grete Raba (grete.raba@gu.se).

#### Materials availability

Unique reagents generated in this study are available from the lead contact with a completed Materials Transfer Agreement.

#### Data and code availability

• The 16S rRNA sequencing data generated during the current study are available in the NCBI Sequence Read Archive and are publicly available as of the date of publication. The dataset identifier is listed in the key resources table. The metaproteome and proteome datasets generated during this study are deposited in the ProteomeXchange Consortium and are available as of the date of publication. Accession numbers for the datasets are listed in the key resources table. The glycomic MS raw files of this study have been deposited in the GlycoPOST database and are publicly available as of the date of publication. The identification number of glycomic dataset is listed in the key resources table.
• This paper does not report original code.
• Any additional information required to reanalyse the data reported in this paper is available from the lead contact upon request.

### Experimental model and study participant details

#### Animal sample collection

Porcine colonic mucins were extracted from flushed porcine colonic tissues, that were acquired from a local butcher (Saaremaa Meat Factory, Estonia) and stored at −20°C until extraction. The mucus layer was gently scraped from the thawed colon epithelium and collected on ice. A total of 138 g of mucosal scrapings were collected and mixed in 1:2 ratio with cold extraction buffer (6 M GuHCl, 5 mM EDTA, 0.01 M NaH_2_PO_4_, pH 6.5) and centrifuged 30 min at 18,000 rpm, 10°C. Floating fat and supernatant were aspirated and mucus pellet was solubilized in 1:1 ratio with cold extraction buffer. The mixture was gently stirred for 3 h at 4°C, after which centrifugation for 30 min at 18,000 rpm and 10°C was repeated. Supernatant was aspirated and the mucus pellet was solubilized in 2:1 ratio with cold extraction buffer. The mixture was stirred gently overnight at 4°C.

The mucus mixture extraction and centrifugation steps were repeated on two consecutive days, after which the mixture was left stirring for 48 h at 4°C. A final centrifugation for 30 min at 18,000 rpm and 10°C was carried out, resulting in 60 g mucus pellet. Freshly prepared reduction buffer [6 M GuHCl, 0.1 M Tris, 5 mM EDTA (ethylenediaminetetraacetic acid), 10 mM DTT (dithiothreitol)] was added to the pellet and stirred gently at 37°C for 5 h. Alkylation was carried out by adding 25 mM IAA (iodoacetamide) to the mixture and stirred gently in the dark for overnight at room temperature. The mucins were dialyzed with 12.4 kDa pore size tubes (Sigma-Aldrich, D0530) against deionized water at 4°C for 24 h, changing the water every 4 h.

#### Human sample collection

Human stool samples were collected from seven healthy adults in Tallinn Estonia. The inclusion criteria were: 19–37 years old, no known active illnesses, ability to provide informed consent and willingness to provide a stool sample. The exclusion criteria were the use of prebiotics, probiotics, laxatives and antibiotics four weeks prior to the sample donation. The donors were male and female, Caucasian. Fecal samples were collected freshly into feces collection tubes (Sarstedt), the tubes inserted into previously frozen cryoblock and kept at −20°C until transport to the lab, but no longer than two days. After arrival to the lab, the samples were stored at −80°C.

The fecal slurries were prepared in an anaerobic chamber (Concept, Baker Ruskinn) flushed with 95% N2. The fecal samples were homogenized in four volumes of sterile PBS (phosphate buffer saline) containing 5% (v/v) DMSO (dimethyl sulfoxide) and freshly autoclaved reducing agent sodium thioglycolate (final concentration 50 mg/mL). Equal volumes of the fecal slurries from different donors were pooled and stored as 0.5 mL aliquots at −80°C until the cultivation experiments.

Collecting and handling the fecal samples was approved by the Tallinn Medical Research Ethics Committee, Estonia (protocol No. 554). All experiments were performed in accordance with relevant guidelines and regulations. Informed consent was obtained from all participants.

### Method details

#### Isothermal microcalorimetry (IMC)

The batch cultivations were carried out in a 48-channel isothermal microcalorimeter (TAM IV, TA Instruments). A chemically defined minimal medium was used to enable quantitative analysis of bacterial metabolism: 0.05 M potassium phosphate buffer was made from 1 M stock solutions (mL/L): K_2_HPO_4_ (28.9) and KH_2_PO_4_ (21.1); mineral salts (mg/L): MgSO_4_∗7H_2_O (36), FeSO_4_∗7H_2_O (0.1), CaCl_2_ (9), MnSO_4_∗H_2_O (3), ZnSO_4_∗7H_2_O (1), CoSO_4_∗7H_2_O (1), CuSO_4_∗5H_2_O (1), (NH_4_)_6_Mo_7_O_24_∗4H_2_O (1), NaCl (527); l-amino acids (g/L): Ala (0.044), Arg (0.023), Asn (0.038), Asp (0.038), Glu (0.036), Gln (0.018), Gly (0.032), His (0.027), Ile (0.060), Leu (0.120), Lys-HCl (0.080), Met (0.023), Phe (0.050), Pro (0.041), Ser (0.095), Thr (0.041), Trp (0.009), Val (0.060), Tyr (0.015); vitamins (mg/L): biotin (0.25), Ca-pantothenate (0.25), folic acid (0.25), nicotinamide (0.25), pyridoxine-HCl (0.50), riboflavin (0.25), thiamine-HCl (0.25), cyanocobalamine (0.25) and other components (g/L): bile salts (0.5), NaHCO_3_ (2.0), Tween-80 (0.5), Na-thioglycolate (0.5), Cys-HCl (0.5, freshly made), hemin (5 mg/L), vitamin K1 (0.5 mg/L).

The substrate screening panel consisted of 14 dietary fibers in combination with commercially available porcine gastric mucin or in-house extracted porcine colonic mucin (Table S2). Each polysaccharide and mucin were added at 2.5 g/L final concentration. The carbohydrate substrates were sterilized separately and mixed with the filter-sterilized medium before cultivation. Culture medium without any added carbohydrates or mucin was used as a negative control. The growth medium was pre-reduced in an anaerobic jar (Anaero-Gen, Oxoid Inc.).

The growth experiments were carried out in sterile hermetically sealed 3 mL ampoules (2 mL working volume, 1 mL headspace). The ampoules were inoculated with 120x dilution of the pooled fecal slurry in an anaerobic chamber (Concept, Baker Ruskinn) and incubated for 64 h at 37°C in isothermal microcalorimeter. Heat flow (P, μW) and total accumulated heat (Q, J) were registered throughout the whole experiment, at 5 min intervals (Table S1).

The ampoules were removed from the calorimeter and the composition of the gas in the headspace was analyzed with gas chromatography. The ampoules were weighed, and the content divided into 0.5 mL aliquots which were centrifuged for 10 min at 10,000 g. The pH of supernatants was measured with pH-meter (InLab Solids, Mettler Toledo). Supernatants were stored at −20°C and pellets at −80°C until further analyses.

#### DNA extraction and sequencing, bioinformatics

DNA was extracted from the cell pellets using PureLink Microbiome DNA extraction kit (Thermo Fisher Scientific). PCR amplification of the V4 hypervariable regions of the 16S rRNA genes was carried out with universal primers F515 5′-GTGCCAGCMGCCGCGGTAA-3′ and R806 5′-GGACTACHVGGGTWTCTAAT-3. Sequencing libraries were prepared with Nextera XT Index Kit (Illumina). Prepared libraries were quantified with Qubit dsDNA HS Assay Kit (quantitation range 0.2–100 ng; Thermo Fisher Scientific) or Qubit dsDNA BR Assay Kit (quantitation range 2–1000 ng; Thermo Fisher Scientific). Pooled libraries were sequenced using Illumina iSeq 100 platform and i1 reagent kit. All reagent kits were handled in accordance with manufacturer’s instructions. The amplified region was 291 bp long and in average 53,616 reads per sample were obtained.

The DNA sequence data was analyzed using BION-meta. Sequences were first cleaned at both ends using a 99.5% minimum quality threshold for at least 18 of 20 bases for 5′-end and 28 of 30 bases for 3′-end. Obtained sequences were then joined and contigs shorter than 250 bp were removed. The sequences were then cleaned of chimeras and clustered by 95% oligonucleotide similarity (k-mer length of 8 bp, step size 2 bp). Consensus reads were aligned to the SILVA ref. 16S rRNA database (v138) using a word length of 8 and similarity cut-off of 90%.

#### Metaproteomics

The microbial cell pellets were dissolved in 60 μL of lysis buffer (4% SDS (sodium dodecyl sulfate), 100 mM Tris-HCl (pH 7.5), 100 mM DTT) and heated at 95°C for 5 min, followed by 2–3 short pulses of ultrasonication (15 μm amplitude). Heating and sonication steps were repeated twice followed by centrifugation for 5 min at 14,000 g to pellet any debris. Cell lysates (30 μL) were digested with LysC and trypsin on 30 kDa cut-off filters (NanoSep, Pall Life Sciences) according to Filter Aided Sample Preparation (FASP) protocol. Peptide yield was measured with a microvolume spectrophotometer (Nano Drop 2000; Thermo Fisher Scientific) at 280 nm wavelength. The samples were acidified with TFA (trifluoroacetic acid) to final concentration of 0.5% and 15 μg of peptides were cleaned and stored in C18-StageTip filters at −20°C.

Samples from three experimental replicates were analyzed in triplicates with an EASY-nLC 1000 system (Thermo Fisher Scientific) connected to a Q-Exactive mass-spectrometer (Thermo Fisher Scientific) through a nanoelectrospray ion source. Peptides were separated with an in-house packed column [150 mm ×0.075 mm inner diameter (New Objective, Woburn), Reprosil-Pur C18-AQ 3 μm particles (Dr. Maisch)] using a gradient: 3–25% B over 175 min, 25–45% B over 30 min, 45–100% B over 5 min and held for additional 20 min on 100% of B, at flow rate of 250 nL/min [A: 0.1% FA (formic acid), B: 80% ACN (acetonitrile), 0.1% FA]. The Q-Exactive HF hybrid quadrupole-Orbitrap mass spectrometer (Thermo Fisher Scientific) was operated at 250°C capillary temperature and 2.0 kV spray voltage. Full mass spectra were acquired in the Orbitrap mass analyser over a mass range from m/z 400 to 1600 with resolution of 60,000 (m/z 200) after accumulation of ions to a 3e6 target value based on predictive AGC from the previous full scan. Twelve most intense peaks with a charge state ≥2 were fragmented in the HCD collision cell with normalized collision energy of 27%, and tandem mass spectrum was acquired in the Orbitrap mass analyser with resolution of 15,000, AGC target value 1e5. Dynamic exclusion was set to 30 s. The maximum allowed ion accumulation times were 20 ms for full MS scans and 50 ms for tandem mass spectrum.

A custom database was constructed for the metaproteomics searches. First, *de novo* peptide identification was done for the raw MS spectra, using PEAKS Studio software (version 8.5, Bioinformatics Solutions Inc). Average local confidence (ALC) was set to be ≥80%. The acquired list of peptides was analyzed with ProteoClade in order to annotate the *de novo* identified peptides to all potential organisms in the sample using the entire UniProt repository.^85^ The list of taxa from ProteoClade was further optimized by i) including only taxa from kingdom of bacteria, ii) removing taxa which were identified in <3 samples per substrate group, iii) adding missing taxa from 16S rRNA gene sequencing analysis results. Protein sequences for each taxon were downloaded from UniProt database (2021.01.14, uniprot.org), reference proteomes preferred where possible. The final custom database contained 290 bacterial as well as pig and human proteomes and was used for the final analysis of MS/MS spectra with MaxQuant (version 1.4). Searches were performed using trypsin as an enzyme, maximum 1 missed cleavage, precursor tolerance of 20 ppm in the first search used for recalibration, followed by 7 ppm for the main search and 0.5 Da for fragment ions. Carbamidomethylation of cysteine was set as a fixed modification and methionine oxidation and protein N-terminal acetylation were set as variable modifications. The required false discovery rate (FDR) was set to 1% both for peptide and protein levels and the minimum required peptide length was set to seven amino acids.

For community composition analysis unique peptides per species were used.

#### Carbohydrate metabolism analysis

All identified proteins were annotated for CAZymes using dbCAN2 (http://bcb.unl.edu/dbCAN2/blast.php). Three tools were used for automatic CAZyme annotation: a) HMMER to search against the dbCAN HMM (Hidden Markov Model) database; b) DIAMOND to search against the CAZy pre-annotated CAZyme sequence database; and c) Hotpep to search against the conserved CAZyme PPR (peptide pattern recognition) short peptide library. To improve annotation accuracy, a filtering step was used to retain only hits to CAZy families found by at least two tools.

#### Porcine mucin analysis

The protein composition of porcine gastric and colonic mucins was analyzed with mass spectrometry system as described for metaproteomics analysis. Briefly, protein samples were digested with trypsin overnight according to Filter aided sample preparation (FASP) protocol.^84^ Peptides were cleaned with C18-StageTip filters and separated with a 45 min gradient of 5–60% buffer B (Buffer B: 80% ACN, 0.1% FA). The raw MS/MS spectra were searched with MaxQuant (version 1.6.11.0) against pig database downloaded from UniProt (2020.09.12).

Composite agarose-polyacrylamide (AgPAGE) gel was prepared according to the protocol of Schulz and co-workers.^83^ Porcine gastric and colonic mucins were solubilized by the addition of 2-times reducing gel-loading buffer (62.5 mM Tris-HCl pH 6.8, 2% SDS, 50 mM DTT 20% (v/v) glycerol) and heated for 5 min at 95°C before separation via AgPAGE for 3.5 h at 30 mA and 6°C and stained with Alcian blue. For controls HiMark Pre-stained Protein Standard (ThermoFisher Scientific, LC5699) and MUC2 purified from LS174T cells (LS material) as described previously^77^ were used.

Porcine gastric mucin was mixed with 5-fold concentrated loading buffer (0.5 M Tris-HCl, pH 6.8, 0.4% (w/v) SDS, 0.5 M EDTA, 0.5 M DTT, 50% (v/v) glycerol) and heated for 5 min at 95°C before separation via SDS-PAGE for 1 h at 150 V and 100 mA and stained with Alcian blue or PAS.

#### Mucin glycan analysis

*O*-glycans were released by reductive beta-elimination at a concentration of 10 mg/mL β-elimination solution (0.5 M NaBH_4_ and 50 mM NaOH). Samples were covered tightly and incubated overnight (ca. 18 h) at 50°C. The samples were acidified with glacial acetic acid (5%, v/v) and desalted using cation exchange resin packed in a C18 ZipTip.

Released glycans were resuspended in water and analyzed by liquid chromatograph-electrospray ionization tandem mass spectrometry (LC-ESI/MS). The oligosaccharides were separated on a column (10 cm × 250 μm) packed in-house with 5 μm porous graphite particles (Hypercarb, Thermo-Hypersil). The oligosaccharides were injected on to the column and eluted with an acetonitrile gradient (Buffer A, 10 mM ammonium bicarbonate; Buffer B, 10 mM ammonium bicarbonate in 80% acetonitrile). The gradient (0–45% Buffer B) was eluted for 46 min, followed by a wash step with 100% Buffer B, and equilibrated with Buffer A in next 24 min. A 40 cm × 50 μm i.d. fused silica capillary was used as transfer line to the ion source.

The samples were analyzed in negative ion mode on an LTQ linear ion trap mass spectrometer (Thermo Electron), with an IonMax standard ESI source equipped with a stainless-steel needle kept at −3.5 kV. Compressed air was used as nebulizer gas. The heated capillary was kept at 270°C, and the capillary voltage was −50 kV. Full scan (*m/z* 380–2000, two microscans, maximum 100 ms, target value of 30,000) was performed, followed by data dependent MS^2^ scans (two microscans, maximum 100 ms, target value of 10,000) with normalized collision energy of 35%, isolation window of 2.5 units, activation q = 0.25 and activation time 30 ms. The threshold for MS^2^ was set to 300 counts. Data acquisition and processing were conducted with Xcalibur software (Version 2.0.7). The LC-MS/MS data was processed using Progenesis QI (Nonlinear Dynamics, Waters).

#### Gas analysis

The microcalorimeter ampoule headspace gas composition was analyzed with a gas chromatograph (Agilent 490 Micro GC Biogas Analyzer, Agilent Technologies Ltd.) using CP-Molsieve 5A and CP-PoraPLOT U columns and a thermal conductivity detector.

#### Analysis of organic acids, free amino acids and amines

The supernatant samples were filtered using centrifugal devices with a 3 kDa cut-off filter (Amicon Ultra-0.5, Merck). The concentrations of organic acids were measured with high-performance liquid chromatography (HPLC) system (Alliance 2795 system; Waters) equipped with BioRad HPX 87H column (Hercules) with isocratic elution of 0.005 M H_2_SO_4_, flow rate 0.5 mL/min, 35°C. RI (model 2414; Waters) and UV (210 nm; model 2487; Waters) detectors were used for quantification with external standards. The data were processed with Empower software (Waters) (Table S1).

The concentrations of free amino acids and amines were determined by the UPLC-UV methodology (Acquity; AccQ·Tag Ultra Derivatization Kit; Waters) developed by Waters with modifications. The standards and samples were derivatised with 6-aminoquinolyl-*N*-hydroxysuccinimidyl carbamate and then loaded on an AccQ-Tag Ultra RP column (130 Å, 1.7 μm, 2.1 × 100 mm; Waters). Amino acids and amines were separated using a gradient of eluent A (AccQ-Tag Ultra eluent A) and eluent B (1% FA in ACN) as follows: 0–0.54 min 99.9% A and 0.1% B. A flow rate of 0.7 mL/min, an autosampler temperature of 8°C, a column temperature of 55°C and injection volume of 1 μL were used. Amino acids and amines were detected with a photodiode array detector (260 nm), and data were processed with Empower 2 software (Waters). The detection limit was 0.001 mM. All standard substrates were of analytical grade.

#### Sequence-based metabolism mapping from metaproteomics

The DNA sequences for enzymes for key metabolic reactions were retrieved from public NCBI database and BLASTed using a local NCBI blastx function. The resulting amino acid sequences were then mapped to the peptides from the metaproteomic analysis. The sum of matching peptides per taxon was considered as protein copy number. The taxa were then divided into similar metabolic groups: 1) Butyric – butyrate producers with 1,2-propanediol capacity (68 taxa), 2) Akkermansia – the main mucin degrader *A. muciniphila* that has a unique metabolism (2 taxa), 3) Bifido – lactate and acetate producers (25 taxa), 4) Bacteroides – propionate/succinate and acetate producers (56 taxa), 5) Enterobacteria – lactate, succinate and acetate producers (12 taxa), 6) Lachnoclostridium – mucin degrading and formate or 1,2-propanediol consumers (18 taxa), 7) Asaccharolytic – lactate and amino acid degraders (6 taxa), 8) Succinivorans – succinate consumers (2 taxa).

### Quantification and statistical analysis

#### Biomass dry weight calculations

The dry weight of each culture was calculated from the total accumulated heat, assuming that roughly 4000 J of heat is released per 1 g of biomass production.

#### Soluble gas concentration calculations

Soluble gas concentration (c) was calculated using Henry law:

$$c=H^{cp}\cdot p$$

where p is the partial pressure of given gas in the gas phase and H^cp∗^ (M/atm) the effective Henry constant of the given gas dependent on pH^86^ (Table S1).

#### Statistical analysis

All statistical analyses were calculated with RStudio version 1.2.5001 (RStudio, Inc., R version R-3-6-1). Alpha diversity (Simpson index) was calculated from taxa identified by the 16S rRNA sequencing. Beta diversity (Bray-Curtis) was calculated from the taxa identified by the 16S rRNA sequencing and the identified proteins by metaproteomics, using the vegan package.^87^ Dunn’s Kruskal-Wallis Multiple comparisons for all conditions were done with the FSA package^88^ (Table S1).

## Supplemental information

Document S1. Figures S1–S4 and Table S2

Table S1. Heatflow, pH values, metabolites concentrations, and statistics, related to Figures 1, 2, 4, S1, and S3 (A) Heatflow at given time points, pH value and metabolites concentrations measured from the end of cultivations. (B) Dunn’s Kruskal-Wallis multiple comparisons for all conditions.

Table S3. MS intensities for protein groups identified by metaproteomic analysis from the end of cultivations, related to Figures 2, 3, and S2–S4 Contains also information of mapping to CAZy and SulfAtlas databases.

Table S4. Enzymes from the end of cultivations identified by sequence-based metabolism mapping from metaproteomic analysis, related to Figure 4 Results are presented as a sum of matching peptides per bacterial group from alignment of enzyme sequence from public NCBI database and metaproteome data.
