# Supplementary material for: Metaproteomics reveals parallel utilization of colonic mucin glycans and dietary fibers by the human gut microbiota
Source: iScience. 2024 May 23;27(6):110093. doi: 10.1016/j.isci.2024.110093 (PMC11214529; doi:10.1016/j.isci.2024.110093)
Supplement: Document S1. Figures S1–S4 and Table S2 [file mmc1.pdf]

iScience, Volume *27*

Supplemental information

Metaproteomics reveals parallel utilization of colonic mucin glycans and dietary fibers by the human gut microbiota

Grete Raba, Ana S. Luis, Hannah Schneider, Indrek Morell, Chunsheng Jin, Signe Adamberg, Gunnar C. Hansson, Kaarel Adamberg, and Liisa Arike

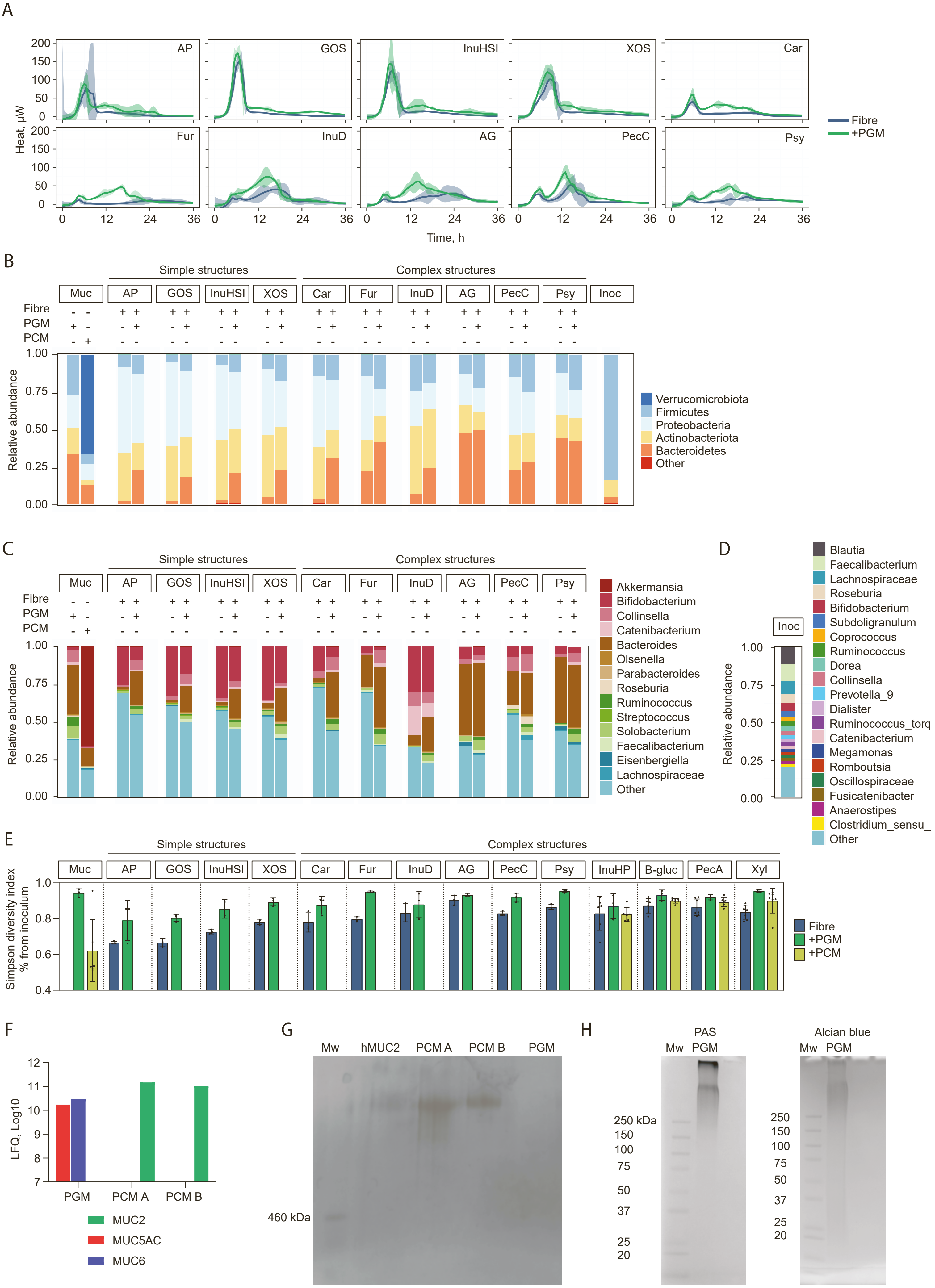
A
Heat, μW
AP
GOS
InuHSI
XOS
Car
Fur
InuD
AG
PecC
Psy
Fibre
+PGM
Time, h
B
Simple structures
Complex structures
Muc
Inoc
Fibre
PGM
PCM
Relative abundance
Verrucomicrobiota
Firmicutes
Proteobacteria
Actinobacteriota
Bacteroidetes
Other
C
Akkermansia
Bifidobacterium
Collinsella
Catenibacterium
Bacteroides
Olsenella
Parabacteroides
Roseburia
Ruminococcus
Streptococcus
Solobacterium
Faecalibacterium
Eisenbergiella
Lachnospiraceae
Other
D
Blautia
Faecalibacterium
Lachnospiraceae
Roseburia
Bifidobacterium
Subdoligranulum
Coprococcus
Ruminococcus
Dorea
Collinsella
Prevotella_9
Dialister
Ruminococcus_torq
Catenibacterium
Megamonas
Romboutsia
Oscillospiraceae
Fusicatenibacter
Anaerostipes
Clostridium_sensu_
Other
E
Simpson diversity index % from inoculum
InuHP
B-gluc
PecA
Xyl
Fibre
+PGM
+PCM
F
LFQ, Log10
PGM
PCM A
PCM B
MUC2
MUC5AC
MUC6
G
Mw
hMUC2
PCM A
PCM B
PGM
460 kDa
H
PAS
Mw PGM
250 kDa
150
100
75
50
37
25
20
Alcian blue
Mw PGM
250
150
100
75
50
37
25
20

Figure S1. The substrate determines the microbiota composition of the faecal consortium, Related to Figure 1. (**A**) Heat evolution of substrate fermentation. The blue line represents the average microbial growth curve on the selected fibre, the green line represents the average microbial growth curve on the selected fibre + porcine gastric mucin (PGM), ± 95% CI, shown as shaded area. n=2-3. (**B**) Phylum-level community composition of the inoculum and the microbiota grown on the selected substrates based on 16S rRNA sequencing. Average relative abundances of the top five phyla. n=2-3. (**C**) Genus-level community composition of the microbiota grown on the selected substrates based on 16S rRNA sequencing. Average relative abundances of the top 14 genera. n=2-3. (**D**) Community composition of the inoculum based on 16S rRNA sequencing. Average relative abundances of the top 20 genera. n=2. (**E**) Simpson alpha diversity indices (±SD) for the panel of tested substrates. n=2-7 (**F**) Gel-forming mucins detected in different mucin samples by mass-spectrometry. MUC2 – UniProt A0A4X1UH57; MUC5AC – UniProt A0A4X1UGK3; MUC6 – UniProt A0A4X1VZC0. (**G**) Mucin samples separated on a composite agarose-polyacrylamide (AgPAGE) gel and stained with Alcian blue. (**H**) PGM sample separated on SDS-PAGE and stained with either PAS or Alcian blue. AP – amylopectin, GOS – galactooligosaccharides, InuHSI – high-soluble inulin, XOS – xylooligosaccharides, Car – κ-carrageenan, Fur – furcellaran, InuD – dahlia inulin, AG – arabinogalactan, PecC – citrus pectin, Psy – psyllium, PGM – porcine gastric mucin, Inoc – inoculum, MUC2 – Mucin-2, MUC5AC – Mucin-5AC, MUC6 – Mucin-6, PCM – porcine colonic mucin.

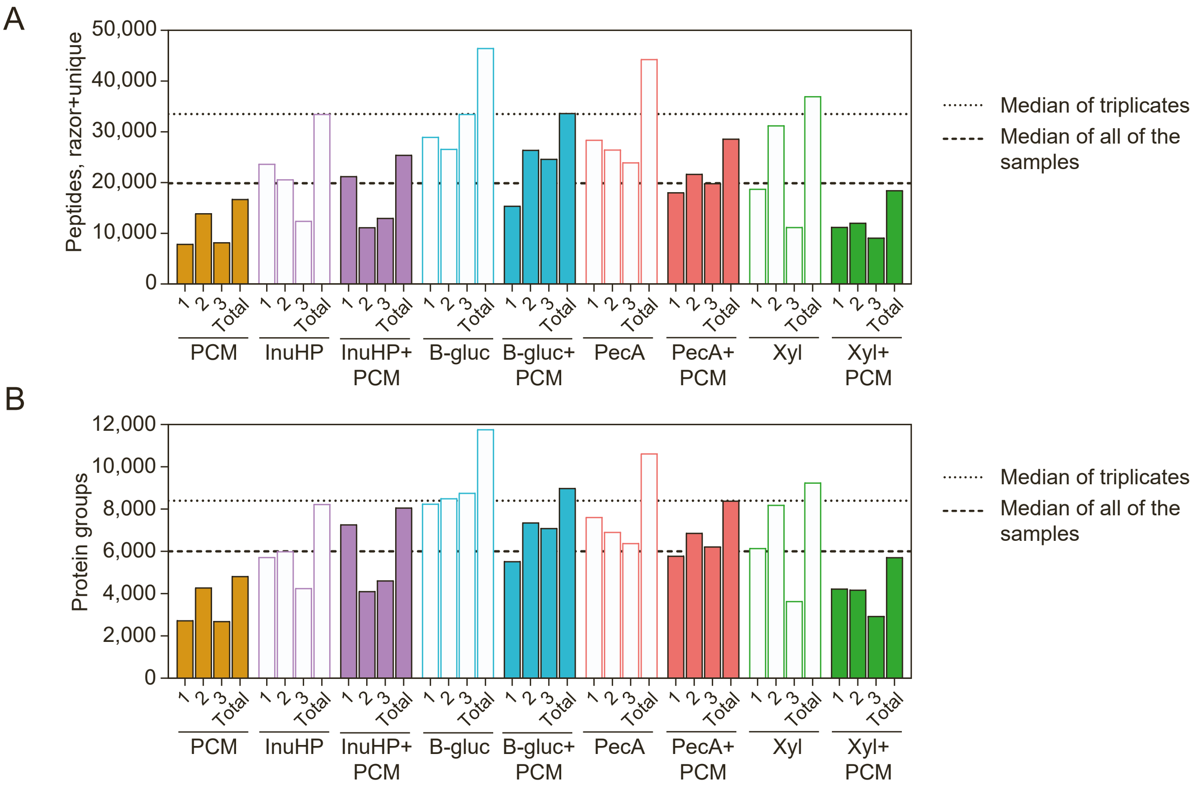

Figure S2. Metaproteomic analysis depth and coverage of proteins related to glycan degradation, Related to Figure 2 and Figure 3. (**A**) The number of peptides identified for each sample. (**B**) The number of protein groups identified for each sample. InuHP – high-performance inulin, B-gluc – β-glucan, PecA – apple pectin, Xyl – xylan, PGM – porcine gastric mucin, PCM – porcine colonic mucin, GH – glycoside hydrolase, CE – carbohydrate esterase, PL – polysaccharide lyase, SusC/D – starch utilization system C or D, CBM – carbohydrate binding module, GT – glycoside transferase.

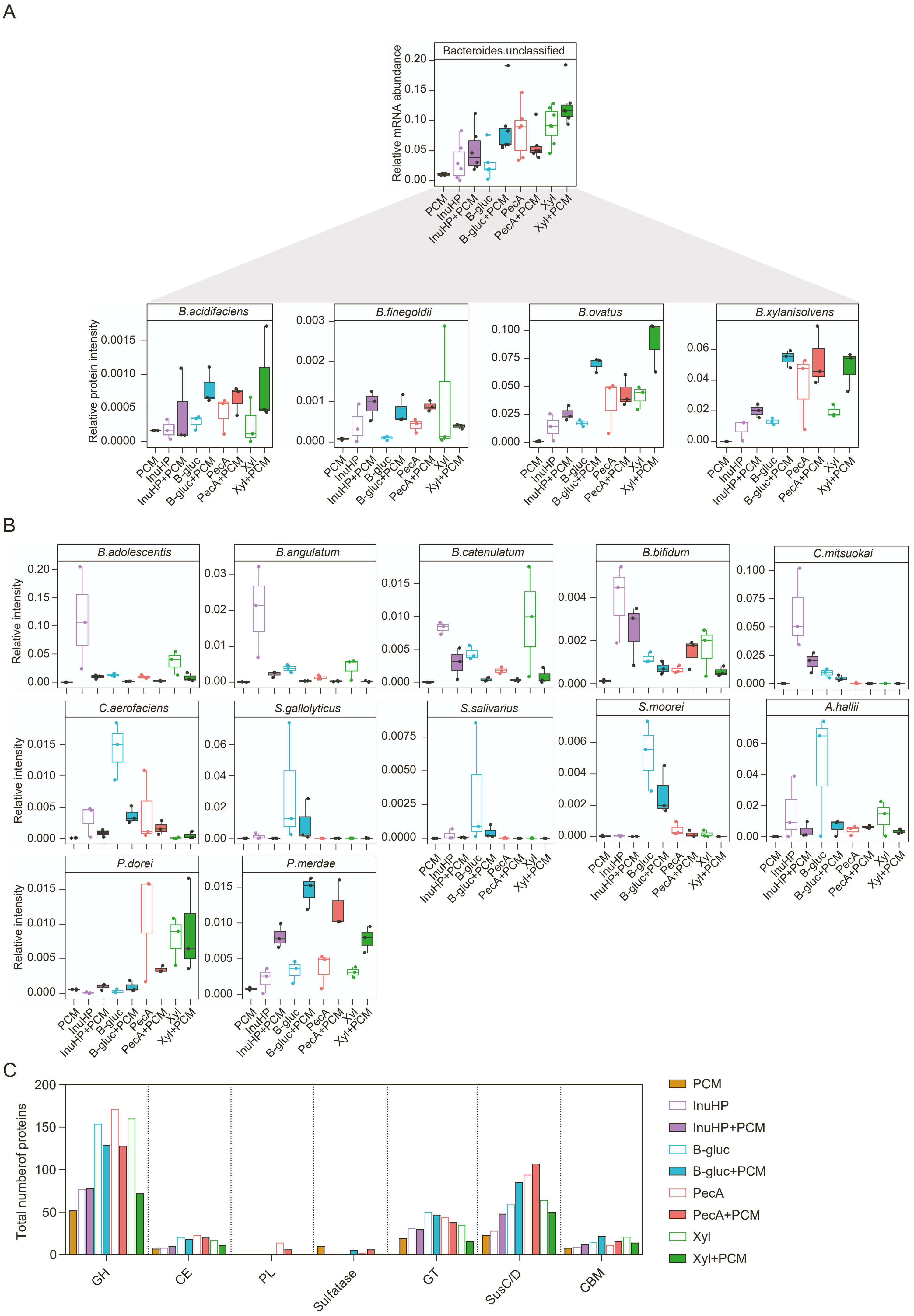
A
Bacteroides.unclassified
Relative mRNA abundance
B.acidifaciens
B.finegoldii
B.ovatus
B.xylanisolvens
Relative protein intensity
PCM
InuHP
InuHP+PCM
B-gluc
B-gluc+PCM
PecA
PecA+PCM
Xyl
Xyl+PCM
B
B.adolescentis
B.angulatum
B.catenulatum
B.bifidum
C.mitsuokai
C.aerofaciens
S.gallolyticus
S.salivarius
S.moorei
A.hallii
P.dorei
P.merdae
Relative intensity
C
Total numberof proteins
GH
CE
PL
Sulfatase
GT
SusC/D
CBM

Figure S3. Metaproteomic analysis of community composition, Related to Figure 2 and Figure 3. (**A**) Comparison of 16S rRNA sequencing and metaproteomic analysis depth. Boxplots showing changes in microbial abundances (relative abundance of 16S rRNA copies or relative intensity of unique peptides) grown on the selected substrates. Colours indicate the choice of substrate, empty boxes represent samples from the cultivation of fibre, filled boxes represent samples from the cultivation of fibre+PCM or sole PCM. Kruskal-Wallis multiple comparisons with Benjamini Hochenberg corrections can be found in Table S1. (**B**) Boxplots showing changes in microbial abundances (relative intensity of unique peptides) grown on the selected substrates. Colours indicate the choice of substrate, empty boxes represent samples from the cultivation of fibre, filled boxes represent samples from the cultivation of fibre+PCM or sole PCM. Kruskal-Wallis multiple comparisons with Benjamini Hochenberg corrections can be found in Supplementary Table S1. (**C**) Total number of proteins mapped to Carbohydrate-Active enZYmes Database (CAZy) and the Database of Sulfatases (SulfAtlas). InuHP – high-performance inulin, B-gluc – β-glucan, PecA – apple pectin, Xyl – xylan, PGM – porcine gastric mucin, PCM – porcine colonic mucin.

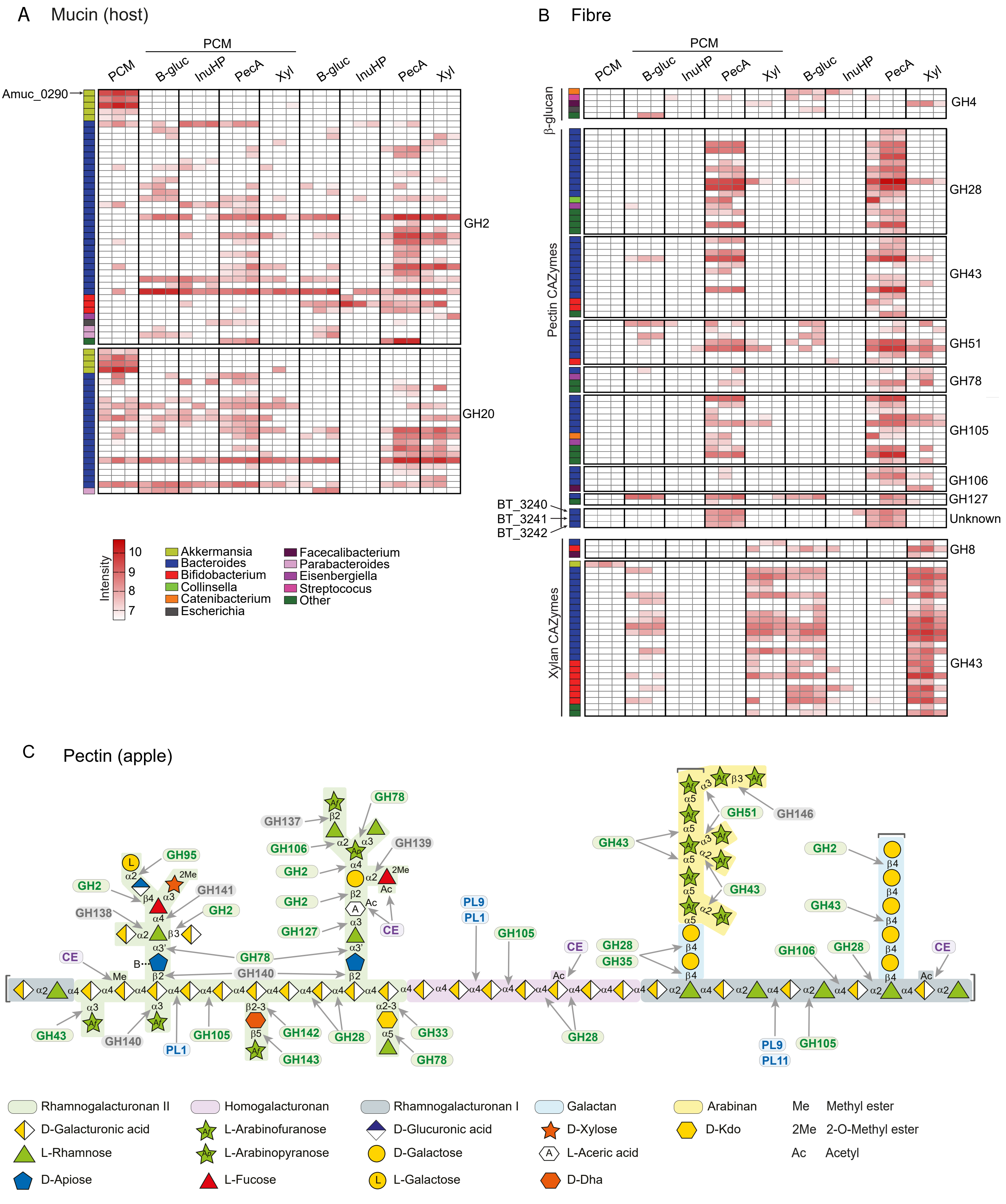

Figure S4. Degradation mechanisms of mucin and fibre by human gut microbiota, Rlated to Figure 3. (**A**) Heatmap of CAZymes targeting mucin *O*-and *N*-glycan linkages. (**B**) Heatmap of CAZymes targeting different fibres. Enzymes grouped by CAZyme families are shown as MS intensity of the protein. For each protein the respective bacterial genus is displayed by a colour code on the left side. (**C**) Pectin structure and the different putative CAZyme families targeting the respective linkages. InuHP – high-performance inulin, B-gluc – β-glucan, PecA – apple pectin, Xyl – xylan, PGM – porcine gastric mucin, PCM – porcine colonic mucin, GH – glycoside hydrolase.

Table S2. Carbohydrate substrates used in IMC cultivation experiments, Related to STAR Methods.

No	Substrate	Physical properties	Abbreviation	Supplier
1	Arabinogalactan from larch wood, Sigma 10830	Water-soluble	AG	Sigma-Aldrich (USA)
2	Amylopectin from maize, Sigma 10120	Water-soluble, gel-forming	AP	Sigma-Aldrich (USA)
3	Beta-glucan from oats	Water-soluble	B-gluc	UNDERSUN BIOMEDTECH (China)
4	Kappa-carrageenan, sulphated plant polysaccharide from *Eucheuma cottonii*, Sigma C1263	Water-soluble, gel-forming	Car	Sigma-Aldrich (USA)
5	Furcellaran, extracted from algae *Furcellaria lumbricalis* (Gigartinales)	Water-soluble, gel-forming	Fur	Est-Agar AS (Kärla, Estonia)
6	Galactooligosaccharides, DP 2-10	Water-soluble	GOS	Friesland Campina (The Netherlands)
7	Inulin from dahlia tubers, Sigma I3754	Water-soluble	InuD	Sigma-Aldrich (USA)
8	High performance inulin, DP >23, 0.1% monosaccharides	Water-soluble	InuHP	Beneo Orafti (Belgium)
9	High soluble Inulin HSI, DP 2-8, 11% monosaccharides	Water-soluble	InuHSI	Beneo Orafti (Belgium)
10	Pectin from apple, Sigma 93854	Water-insoluble	PecA	Sigma-Aldrich (USA)
11	Pectin from citrus peel, Galacturonic acid >74%, Sigma P9135	Water-insoluble	PecC	Sigma Aldrich (USA)
12	Psyllium (Carepsyllium), from *Plantago ovatas,* 80 %	Water-insoluble, gel-forming	Psy	Caremoli (Italy)
13	Xylooligosaccharides, 80 %	Water-soluble	XOS	Anhui Elite Ind Co (China)
14	Xylan from beechwood, Sigma X4252	Water-insoluble	Xyl	Sigma-Aldrich (USA)
15	Mucin from porcine stomach, Type III, Sigma M1778	Water-insoluble, gel-forming	PGM	Sigma-Aldrich (USA)
16	Mucin from porcine colon	Water-insoluble, gel-forming	PCM	This study
